# Associations of Caregiver-Reported Unmet Needs and Burden-Related Indicators With Excellent Well-Being: A Cross-Sectional Study

**DOI:** 10.1177/00469580261466521

**Published:** 2026-07-06

**Authors:** Sirrý Sif Sigurlaugardóttir, Sigurveig H. Sigurðardóttir, Thor Aspelund, Kristín Björnsdóttir, Magnus Jegermalm, Kjartan Ólafsson, Ingibjörg Hjaltadóttir

**Affiliations:** 1 Faculty of Social Work, 275019 University of Iceland, Reykjavik, Iceland; 2 Faculty of Medicine, 275018 University of Iceland, Reykjavik, Iceland; 3 Faculty of Nursing and Midwifery, 275018 University of Iceland, Reykjavik, Iceland; 4 Department of Social Sciences, 531597 Marie Cederschiöld University, Stockholm, Sweden; 5 Social Science Research Institute, 63541 University of Iceland, Reykjavik, Iceland

**Keywords:** informal caregivers, caregiver well-being, caregiver burden, unmet needs, interRAI-SCaN, cross-sectional

## Abstract

**Introduction:**

Informal caregivers provide essential assistance to older adults living at home, but can experience burden and unmet needs. While caregiver burden is well studied, its conceptual boundaries and relationship to unmet needs remain unclear. This cross-sectional study examined the associations of caregiver-reported unmet needs and burden-related indicators with excellent well-being among informal caregivers.

**Methods:**

A cross-sectional survey was conducted among 352 informal caregivers of older adults receiving home nursing services in Iceland, using the interRAI Self-Reported Carer Needs (SCaN) assessment. Caregiver well-being was dichotomized into excellent and non-excellent. A hierarchical logistic regression model was used to examine associations of caregiver-reported unmet needs and burden-related indicators with excellent well-being.

**Results:**

The most frequently reported caregiver unmet needs concerned episodic relief (44.5%), psychological counseling (31.6%), and health education (30.5%). In the final hierarchical model, caregiver-reported care-recipient unmet need for housing adaptation (OR = 0.42, 95% CI [0.19–0.92]) and caregiver unmet need for psychological counseling (OR = 0.44, 95% CI [0.22–0.85]) were associated with lower odds of excellent well-being. Decreased social activities due to caregiving (OR = 0.47, 95% CI [0.24–0.95]), perceiving caregiving as a source of stress (OR = 0.37, 95% CI [0.19–0.72]), and higher multifaceted strain (OR = 0.87, 95% CI [0.81–0.94]) were also associated with lower odds of excellent well-being.

**Conclusions:**

Caregiver-reported unmet needs and burden-related indicators were associated with excellent caregiver well-being. Systematic caregiver assessments may help identify both support gaps and subjective strain, informing targeted support for informal caregivers.

## Introduction

An aging population requires adjustments in service delivery, particularly in how health and social care systems support both care recipients and informal caregivers. Societies must update and re-evaluate these systems to ensure that services are timely and appropriate. Research and policy efforts increasingly reflect this direction, drawing on empirical evidence that highlights the interdependence between care recipients and informal caregivers. As part of the European Pillar of Social Rights, *the European Care Strategy* was launched to enhance the resilience of services for care recipients and caregivers,^1^ establishing a policy framework to improve access to affordable, sustainable long-term care while recognizing the role of informal caregivers in the European system. Similar efforts in Canada reflect recognition of this dual focus.^2,3^

Family caregivers and other informal caregivers play a vital role in geriatric care worldwide,^4^ yet usually receive no compensation and limited training.^5^ The literature documents caregiver burden,^6^ unmet needs,^7^ the lack of universal measurements for assessing service needs,^8^ and the limited availability of interventions within health and social care systems.^9,10^ In this context, unmet needs can be understood as a mismatch between the support caregivers require and the support they actually receive. Research has shown that such unmet needs are closely linked to both caregiver burden and well-being.^9,11^

Previous research has identified a range of unmet needs among informal caregivers, including emotional support, information and education, respite from caregiving responsibilities, and assistance with navigating health and social care systems.^7,9^ Similarly, caregiver burden has been conceptualized as a multidimensional construct encompassing emotional, physical, social, and financial strain associated with the caregiving role.^6,10^ These dimensions of burden, together with unmet support needs, have been shown to influence caregiver well-being, with higher levels of unmet needs and burden associated with poorer psychological health and reduced quality of life.^9,11^ Despite this, unmet needs are often not examined alongside burden-related indicators, limiting understanding of caregivers’ support needs.

Many caregivers are well-equipped to manage caregiving with minimal support, whereas others struggle and experience negative impacts on daily life, sometimes increasing the care recipient’s need for official services.^12^ Beyond practical implications, supporting caregivers is important to protect their health and well-being and sustain caregiving over time. It is therefore essential to identify caregivers who may be struggling or at risk of reduced well-being, and to consider how their needs can be met. Reflecting this, there is growing interest in viewing the caregiver and care recipient as a dyad within the service system, as support for one supports the other.^11,13^ Suggestions have been made to classify informal caregivers as service users themselves or as part of the caregiving team.^11^ However, such needs and experiences may differ between types of caregivers.

Previous research suggests that caregivers may require different kinds of support^14^ depending on their relationship with the care recipient^15,16^ and living arrangements.^17^ Spousal caregivers are often described as the most burdened^18,19^ and the most likely to provide care without help from others.^18,20^Non-cohabiting caregivers can still be heavily involved, but they typically share responsibility within a broader “care network” that includes informal supporters and formal eldercare services.^20-23^ Such differences in arrangements may influence burden-related experiences and support needs.

Providing informal care can have consequences that may affect caregivers’ ability to retain employment, including health issues, exhaustion, sleep deprivation, and financial difficulties. Reducing paid work hours can further exacerbate concerns and increase caregivers’ own support needs.^24^

While caregiver burden and well-being have been widely studied, less attention has been paid to how unmet needs are considered alongside burden-related indicators. Existing research has often examined caregiver outcomes without systematically addressing whether caregivers’ needs are met. Understanding support needs and identifying those at higher risk through appropriate screening may improve assessment and support.

This cross-sectional study examines caregiver-reported unmet needs and burden-related indicators in relation to excellent well-being among informal caregivers of older adults receiving home care nursing in Iceland’s capital region. Specifically, it asks: What unmet needs are most commonly reported by informal caregivers? How are selected unmet needs and burden-related indicators associated with excellent caregiver well-being?

### Theoretical Framework

This study is informed by Pearlin et al.’s stress process model, which conceptualizes caregiving as a process shaped by interactions among background characteristics, stressors, resources, appraisal, and outcomes.^25^ Within this broader lens, caregiver burden is understood through Liu et al's^10^ conceptual framework, which defines caregiver burden as “*the level of multifaceted strain perceived by the caregiver from caring for a family member and/or loved one over time” (p*.*442)*. This framework specifies burden as a multidimensional construct involving physical, emotional, social, relational, and economic challenges.^10^ In the present study, caregiver-reported unmet needs are incorporated as support-related indicators that may coexist with burden-related experiences in relation to excellent caregiver well-being.

More specifically, caregiver-reported unmet needs are conceptualized as support-related gaps arising when needed support is unavailable, inaccessible, insufficient, or not perceived as adequate to the situation. These unmet needs include caregivers’ own support needs and their assessments of unmet service or support needs for the care recipient. Within Pearlin et al.’s stress process model,^25^ unmet needs can be understood as resource gaps that may function as contextual stressors, constraining coping capacity and shaping appraisal of the caregiving role. They are therefore examined as support-related gaps alongside burden-related indicators.

Building on this perspective, caregiving is seen as a dynamic process shaped by continuous negotiation between demands, resources, and coping capacity. Caregiver burden and well-being have been conceptualized as interdependent and reciprocally influential^26^; increased burden is associated with a decline in well-being, whereas protective factors may alleviate burden. Caregivers may experience both burdens and positive aspects of caregiving simultaneously, underscoring the need to examine caregiving in its broader context.^27^

Maslow^28^ defined a human need as a state of deficiency motivating behavior aimed at fulfillment. The concept has since been expanded across contexts, including Bradshaw’s^29^ typology of needs and more recent classifications of caregivers’ needs, distinguishing enabling domains and direct support needs.^30^ Needs can therefore be understood both from the perspective of those experiencing them and in relation to the support available to meet them.

To conceptualize unmet needs, this study draws on Bradshaw’s^29^ typology of needs, with emphasis on *felt* and *expressed needs*. Felt needs reflect individuals’ internal perception of lack, whereas expressed needs are actively stated, for example, through survey responses. In this study, caregivers assessed both their own support needs in daily life and the care recipient’s needs. These assessments included specific areas such as participation in a carer support group, psychological counseling, health education, episodic relief, financial or legal advice, and transportation assistance, as well as care recipient needs, including housing adaptation, delivered meals, physical therapy, daycare services, personal care, and household tasks.^31^ The data, therefore, reflect caregivers’ subjective evaluations of whether each need was fully met, partially met, or unmet, thereby capturing caregivers’ perceptions of need fulfillment and of the perceived responsiveness of available support systems.

By focusing on expressed needs, the study centers caregivers’ perspectives in examining unmet needs alongside burden-related indicators in relation to excellent well-being. It furthers the ongoing discussion of how to evaluate informal caregivers’ situations, identify indicators of concern, and provide support that meets identified needs.^10,32,33^ Together, these perspectives provide the conceptual foundation for examining caregiver-reported unmet needs alongside burden-related indicators in relation to excellent caregiver well-being.

## Methodology

### Study Design and Population

A cross-sectional survey examined the associations of caregiver-reported unmet needs and burden-related indicators with excellent well-being among informal caregivers in four municipalities in Iceland’s capital area. Icelandic society is highly homogeneous, and welfare services are delivered within a state-centered, publicly funded, universal coverage model.^34^ A total population approach was applied; all named caregivers of service users at the time were eligible for participation. Caregivers were eligible if they provided care to older people aged ≥65 years who had been receiving home care nursing services for at least three months. Home care nursing staff asked each care recipient to identify their primary caregiver (who provided the greatest level of support) and, where applicable, a secondary caregiver.

Eligible participants were those aged ≥18 years, proficient in Icelandic, and without impairments that could hinder participation, e.g., self-reported mental illness or cognitive impairment. Participants could complete the survey only once and were asked to refer to a single care recipient, even if they cared for more than one person (e.g., both parents). Exclusion criteria included non-Icelandic speakers and individuals who were already residing in long-term care at the time of recruitment. Home care nursing staff contacted caregivers by telephone, informed them about the study, and obtained verbal permission to send the survey link. Electronic informed consent was obtained prior to data collection.

A secure survey link was subsequently sent via text message. Participants unable or unwilling to complete the online survey could respond by phone or in person.

Of the 691 caregivers contacted, 483 (70%) accessed the survey. Those who did not attempt to log in within two weeks were contacted by phone. Non-respondents were 208 (30%), including 24 (3%) who declined, and 184 (27%) were unreachable. Analyses were restricted to cases with valid responses to the outcome variable, the Caregiver Well-Being Index (n = 352). For regression analyses, missing data were handled using listwise deletion, and the full participation flowchart is presented in Table S1.

Ethical approval was granted by the Icelandic Ethical Review Committee (VSN-21-170) and the Scientific Committee of the Health Care Center of the Capital Area (14.01.22).

### Measures

Data were collected using the interRAI Self-Reported Carer Needs (SCaN) Assessment, which assesses informal caregivers’ daily lives and caregiving roles. InterRAI is a global non-profit network that develops standardized assessment tools for health and social care.^31^ The original English version was translated into Icelandic by five members of the research team. A back-translation was conducted by an independent bilingual team and approved by the interRAI instrument authors.

*Caregiver well-being* was assessed using the Caregiver Well-Being Index (CWBI), a validated four-item screening tool embedded within the interRAI SCaN,^35^ designed to measure psychological well-being by capturing emotional strain experienced over the previous three days, including depressive symptoms and anxiety. Items are scored on a scale of 0 to 2, yielding a total score of 0 to 8. Based on established thresholds, participants were categorized into four levels of well-being: excellent (0), good (1–3), fair (4–6), and poor (7–8), with higher scores indicating lower psychological well-being.^35^ In this study, the CWBI served as the primary outcome variable to examine factors associated with excellent caregiver well-being. Accordingly, the outcome variable was dichotomized into excellent well-being (score = 0) versus non-excellent well-being (scores 1–8), reflecting an analytic focus on factors associated with the highest level of recent caregiver psychological well-being.

*Unmet needs* were measured using selected items from relevant interRAI SCaN sections. These sections address caregivers’ functional ability and support needs, as well as caregiver-reported support needs of the care recipient. For each domain, participants reported whether support was needed and whether it was adequately met. A need was considered unmet if no support was provided, or if the support provided was insufficient. The same approach was used to identify unmet needs among care recipients, based on the caregiver’s assessment. Time frames varied across domains, ranging from the past three days to general or ongoing needs. The items and variables correspond to other interRAI instruments; the time frames, therefore, follow established standards.^36^ However, as recall periods differ across domains, this may limit direct comparability between items. Each unmet need was recoded as a separate binary variable. All items included in the unmet needs measure are presented in Table 2 and Supplementary Table S2.

*Burden-related indicators* were measured using selected interRAI-SCaN items informed by Liu et al.’s^10^ conceptual framework of caregiver burden. The selected variables captured burden-related conditions and experiences, including social restriction, caregiving appraisal, and multifaceted strain. The selection and operationalization of these variables have been described previously.^37^ In line with interRAI guidance, the validated wording of the interRAI-SCaN is not reproduced; instead, the instrument is referenced, and items are described using simplified or paraphrased formulations.^31^

### Statistical Analysis

Descriptive statistics summarize the caregivers’ sociodemographic characteristics, unmet needs, and well-being scores. Analyses were restricted to cases with valid Caregiver Well-Being Index (CWBI) responses (maximum n=352). The sample size varied slightly due to item-level missing data (<1%). Cross-tabulations with chi-square tests examined bivariate relationships between excellent caregiver well-being and key independent variables, including demographics and individual unmet needs.

Univariate logistic regression identified factors associated with excellent caregiver well-being. The CWBI was dichotomized into excellent well-being (score = 0) and non-excellent well-being (scores 1–8), with higher scores indicating poorer well-being. For regression analyses, the dichotomized outcome was coded as excellent well-being = 1 and non-excellent well-being = 0. Each predictor was entered separately to assess its association with excellent caregiver well-being. Odds ratios (OR) and 95% confidence intervals (CI) were reported. Full univariate results are shown in Table S5. Variables for the hierarchical model were selected based on preliminary analyses and theoretical relevance.

A hierarchical multivariable logistic regression model was estimated using a blockwise enter method, with cumulative steps reported as Models 1–4. Complete cases for variables included in the hierarchical model were used (n = 347). Variables were entered cumulatively in four blocks: background characteristics (Model 1), selected unmet needs (Model 2), social restriction (Model 3), and caregiving appraisal and strain (Model 4). The ordering of blocks reflected the study’s aims and was informed by Pearlin et al.’s^25^ stress process model. Model improvement was assessed using block chi-square tests and Nagelkerke R^2^, and model fit was assessed using the Hosmer–Lemeshow test.

All analyses were conducted in IBM SPSS Statistics version 30.0.0.0(172), with statistical significance set at *p* < 0.05. A post hoc power analysis was conducted using G*Power based on observed effect sizes (Cohen’s w) from key chi-square analyses. This study was reported in accordance with the STROBE guidelines for observational studies,^38^ and a completed checklist is included as a supplementary file (Table S3).

## Results

### Descriptive Statistics

Table 1 shows that among caregivers, 66.2% (233) reported excellent well-being, whereas 33.8% (119) reported good, fair, or poor well-being. The mean CWBI category score was 1.57 (SD = 0.9, range 1-4). Cronbach’s alpha^39^ was 0.892, with corrected item-total correlations ranging from 0.693 to 0.805. Alpha did not increase with the deletion of any item.

**Table 1.** Demographic Distribution of the Sample (n = 352)

Variable		%
**Caregiver**		
age		
49 or younger	16.2	
50 - 64	40.6	
65 - 74	27.0	
75 or older	16.2	
female	68.5	
primary caregiver	59.9	
**Care recipient**		
Age		
65 – 74	14.5	
75 – 84	33.5	
85 or older	52.0	
Female	59.9	
**Caregiver relationship to care recipient**		
Spouse/partner	19.6	
Child/child-in-law	74.7	
Other	5.5	
**Caregiver marital status**		
Married/cohabiting	82.1	
Single	17.6	
**Caregiver travel time to care recipient**		
We live together	22.7	
1-14 minutes	46.3	
15-29 minutes	23.9	
30-59 minutes	2.3	
60 minutes+	4.5	
**Length of caregiving**		
Less than 1 year	8.0	
1 - 5 years	49.1	
More than 5 years	42.6	
**Caregiver well-being level (CWBI)**		
Excellent well-being	66.2	
Good well-being	18.2	
Fair well-being	8.5	
Poor well-being	7.1	

**Figure 1.**
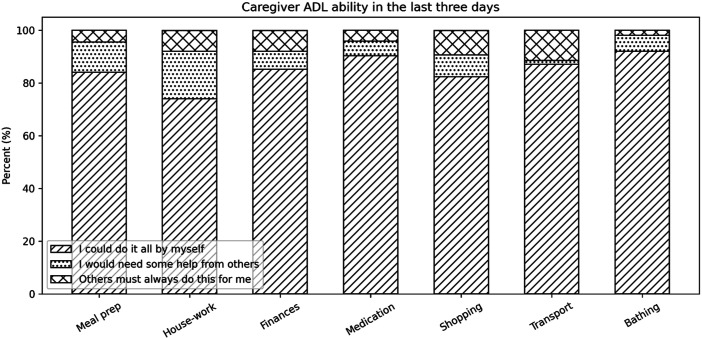
Caregiver-reported functional ability in the last three days (n = 350)

Figure 1 and Table S2 show that independence ranged from 74.7% (263) in household chores to 92.3% (325) in bathing.

Assistance was most often needed for housework, while complete dependence was most frequently reported for transportation (9.9%; 35) and shopping (8.2%; 29). Reliability of the seven-item ADL scale was high (α = 0.900; item–total correlations 0.628–0.817), with no item affecting the alpha when removed.

Table 2 shows care-recipient support needs as reported by caregivers, based on 14 service areas that capture both services currently received and those the caregiver believed were needed. Distributions varied considerably across services. The Cronbach’s alpha^39^ for these items was 0.769, and corrected item-total correlations ranged from 0.113 to 0.480. Alpha did not increase if any item was deleted.

**Table 2.** Met and Unmet Needs (n = 348)

Care recipient met and unmet needs according to the caregiver				
Type of service	Not received, not needed	Received, no more needed	Not received, but needed	Received, but more needed
	Met needs		Unmet needs	
	%	%	%	%
Personal care	14.5	28.1	14.8	41.5
Household tasks	8.0	34.1	11.6	45.2
Medical or nursing	7.4	39.5	13.6	38.4
Mental health	40.1	7.1	40.3	11.4
Delivered meals	34.4	29.5	23.9	11.1
Physical therapy	15.3	23.6	34.4	25.6
Daycare services	38.6	13.1	30.7	16.5
End-of-life care	89.8	2.8	3.7	2.6
Housing adaptation	65.9	15.6	9.7	7.7
Communication	89.5	1.1	6.5	1.7
Assistive devices	13.9	51.7	10.5	22.7
Transportation	36.6	28.1	19.3	14.8
Financial or legal	83.2	8.2	5.1	2.3
Educational support	95.2	0.9	1.7	1.1

Self-reported needs of the caregiver				
Type of service	Not received, not needed	Received, no more needed	Not received, but needed	Received, but more needed
	Met needs		Unmet needs	
	%	%	%	%
Carer support group	71.0	3.7	23.3	2.0
Psychological counseling	67.0	1.4	28.4	3.2
Health education	67.2	2.3	27.9	2.6
Episodic relief	50.9	4.6	27.3	17.2
Financial/legal advice	90.5	1.4	6.6	1.4
Transportation assistance	82.2	5.5	8.6	3.7

Table 2 also shows caregivers’ own support needs, based on services they received and those identified as needed. Variation was observed across services. For example, 71.0% (247) reported neither receiving nor needing support groups, while 23.3% (81) indicated a need for such support. However, 90.5% (315) reported not receiving financial or legal advice, with 6.6% (23) identifying a need for it. Cronbach’s alpha^39^ for these items was 0.700, and corrected item-total correlations ranged from 0.342 to 0.531. Alpha did not increase if any item was deleted.

Cross-tabulations and chi-square tests were used to examine differences in the prevalence of unmet needs across groups defined by sociodemographic and caregiving-related variables; results are presented in Table S4.

### Regression Analysis

Table 3 presents a hierarchical logistic regression model of excellent caregiver well-being based on 347 complete cases. Background characteristics were entered in Model 1; selected unmet needs in Model 2; social restriction in Model 3; and caregiving appraisal and strain in Model 4. Each block was statistically significant (p < 0.001), Nagelkerke R^2^ increased from 0.15 to 0.51, and the final model showed acceptable fit (Hosmer–Lemeshow p = 0.44).

**Table 3.** Variables Included in Hierarchical Logistic Regression Models of Excellent Caregiver Well-Being (n = 347)

Variable	Model 1	Model 2	Model 3	Model 4
**Background characteristics**				
Female caregiver	0.41 [0.24–0.71]*	0.44 [0.23–0.83]*	0.42 [0.22–0.80]*	0.65 [0.32–1.31]
Caregiver age, years	1.03 [1.00–1.06]	1.00 [0.97–1.04]	1.01 [0.98–1.05]	1.02 [0.98–1.06]
Married/cohabiting caregiver	2.92 [1.61–5.31]*	2.61 [1.33–5.12]*	2.44 [1.20–4.95]*	2.34 [1.12–4.88]*
Care recipient age, years	0.96 [0.92–1.00]*	0.96 [0.91–1.00]	0.95 [0.90–0.99]*	0.93 [0.88–0.98]*
Child/child-in-law caregiver	5.86 [2.45–14.03]*	4.17 [1.59–10.98]*	4.72 [1.77–12.57]*	4.59 [1.64–12.85]*
**Unmet needs**				
CG unmet needs with shopping assistance	​	0.27 [0.13–0.53]*	0.36 [0.18–0.73]*	0.45 [0.20–1.00]
CG-reported CR unmet need for housing adaptation	​	0.26 [0.13–0.52]*	0.28 [0.13–0.58]*	0.42 [0.19–0.92]*
CG unmet need for psychological counseling	​	0.19 [0.10–0.34]*	0.26 [0.14–0.49]*	0.44 [0.22–0.85]*
**Social restriction**				
Decreased social activities due to caregiving	​	​	0.29 [0.15–0.55]*	0.47 [0.24–0.95]*
**Caregiving appraisal and strain**				
Caregiving as a source of stress	​	​	​	0.37 [0.19–0.72]*
Multifaceted strain	​	​	​	0.87 [0.81–0.94]*
**Model statistics**				
Block χ^2^(df)	39.97 (5)*	75.68 (3)*	15.18 (1)*	27.68 (2)*
Nagelkerke R^2^	0.15	0.39	0.44	0.51
Hosmer–Lemeshow p	0.12	0.97	0.91	0.44

*Note.* Odds ratios (Exp(B)) with 95% confidence intervals in brackets. * Indicates statistical significance at p < 0.05. CG = caregiver; CR = care recipient.

In the final model, married/cohabiting caregivers (OR = 2.34, 95% CI [1.12–4.88]) and child/child-in-law caregivers (OR = 4.59, 95% CI [1.64–12.85]) had higher odds of excellent well-being. Each additional year of care-recipient age was associated with lower odds of excellent well-being (OR = 0.93, 95% CI [0.88–0.98]). Caregiver-reported care-recipient unmet need for housing adaptation (OR = 0.42, 95% CI [0.19–0.92]) and caregiver unmet need for psychological counseling (OR = 0.44, 95% CI [0.22–0.85]) remained associated with lower odds of excellent well-being. The caregiver’s unmet need for shopping assistance was not statistically significant in the final model (OR = 0.45, 95% CI [0.20–1.00], p = 0.051).

Decreased social activities due to caregiving (OR = 0.47, 95% CI [0.24–0.95]), perceiving caring as a source of stress (OR = 0.37, 95% CI [0.19–0.72]), and each one-point increase in multifaceted strain (OR = 0.87, 95% CI [0.81–0.94]) were associated with lower odds of excellent well-being.

## Discussion

This study examined associations of caregiver-reported unmet needs and burden-related indicators with excellent well-being among informal caregivers of older adults receiving home nursing. The findings showed that unmet needs were common and diverse, with episodic relief, psychological counseling, and health education among the most frequently reported caregiver support needs. In the final model, caregiver-reported care-recipient unmet need for housing adaptation and caregiver unmet need for psychological counseling remained associated with lower odds of excellent well-being after burden-related indicators were added. Married/cohabiting caregivers and child/child-in-law caregivers had higher odds of excellent well-being, whereas older care-recipient age was associated with lower odds. Decreased social activities, perceiving caregiving as a source of stress, and higher multifaceted strain were also associated with lower odds of excellent well-being. These findings suggest that systematic assessment of both unmet needs and burden-related indicators may help identify caregivers who could benefit from additional support.

The majority of caregivers were women (68.5%), consistent with an international review indicating that around 70% of caregivers are female.^40^ The most frequently reported caregiver-assessed unmet needs among care recipients concerned physical therapy, household tasks, personal care, and medical or nursing services, suggesting that core home and rehabilitation services may not always be adequate. These findings align with the concept of *care poverty,* which describes the gap between care needs and available resources.^41^

Caregivers commonly reported unmet support needs related to psychological counseling, health education, and episodic relief, with the highest level observed for episodic relief. These findings indicate persistent gaps in emotional support and episodic relief, consistent with evidence that self-care needs, particularly stress management, are often unmet^42^ and that access to educational and episodic relief services remains limited.^11^ Assessment of caregivers’ functional ability showed that most were independent, though some required assistance with instrumental tasks such as transportation and shopping. ADL assessments have traditionally been conducted by professionals, sometimes relying on care recipients’ self-reports. More recently, caregiver-reported tools have been introduced to evaluate older adults’ functional status.^43^ This study demonstrates how the interRAI-SCaN can be used to assess caregivers’ own functional capacity. Caregivers’ own functional capacity may therefore be relevant when assessing support needs.

Patterns of unmet needs varied by caregiver characteristics. Female caregivers more often reported unmet needs for psychological counseling and episodic relief, and perceived unmet mental health needs in care recipients. These findings align with evidence that women carry a disproportionate share of emotional and support-related caregiving demands.^40^ Age differences were also evident. The oldest caregivers (75+) reported unmet needs for assistance with instrumental activities of daily living more often than others, including housework, shopping, finances, medication, and transportation. This aligns with qualitative findings showing that informational gaps and delayed self-care are common among older caregivers.^7^ In contrast, younger caregivers more often reported care-recipient unmet service needs and their own needs for psychological counseling and financial or legal advice. These findings are consistent with those of the Belgian Health Interview Survey, which showed higher psychological distress among younger caregivers.^44^

Differences by gender and age may reflect both structural inequalities and the relational and moral context of caregiving. Informal caregiving is grounded in a private, explicit, mutual agreement between individuals, an understanding that “this is something we both want” or “this is something we do together.” At its core, it concerns relationships between individuals, which may involve reciprocity and moral dimensions, such as willingness to assume responsibility.^45,46^ These relational and ethical aspects of caregiving help explain variations in unmet needs and raise the question of how these needs are linked to well-being.

The hierarchical model showed that selected unmet needs were associated with excellent well-being alongside burden-related indicators. Caregiver-reported care-recipient unmet need for housing adaptation remained associated with lower odds of excellent well-being, aligning with evidence that housing adaptations can affect informal caregivers’ everyday lives and burden.^47^ Caregiver unmet need for psychological counseling also remained associated with lower odds of excellent well-being, consistent with evidence on the importance of mental health support for caregivers, including telephone-delivered cognitive behavioral therapy^48^ and acceptance and commitment therapy for dementia caregivers.^49^ Caregiver unmet need for shopping assistance was attenuated and did not reach statistical significance in the final model, suggesting possible overlap with broader caregiving demands and burden-related experiences.

The burden-related indicators also remained important. Decreased social activities due to caregiving, perceiving caregiving as a source of stress, and higher multifaceted strain were each associated with lower odds of excellent well-being. These findings are consistent with Pearlin et al.’s stress process model^25^ and the caregiver burden framework by Liu et al,^10^ where caregiving is understood as a process shaped by stressors, resources, subjective appraisal, and consequences over time. They also align with recent evidence showing that subjective burden and reduced social engagement are associated with poorer psychological well-being among family caregivers.^50^ The findings suggest that excellent caregiver well-being is associated with both external service gaps and subjective caregiving strain. Unmet needs should therefore not be treated only as consequences of burden, but as support-related indicators that may coexist with burden-related experiences and help identify caregivers at risk of reduced well-being.

### Implications for Policy and Practice

Findings indicate that unmet needs and burden-related indicators should be assessed together when identifying caregivers who may require support. Current policies often acknowledge informal caregivers but rarely include approaches for assessing and meeting their needs.^41^ The results highlight the importance of integrating caregiver assessments into health and social care practice, including assessments of caregivers’ functional ability, psychological support needs, social restrictions, and perceived strain.

Policy efforts may need to prioritize accessible psychological counseling, episodic relief, and practical support such as housing adaptation. Although episodic relief was not included among the final unmet need indicators in the hierarchical model, it was among the most frequently reported unmet needs and remains relevant for service planning. In practice, caregiver assessments should capture both caregivers’ own support needs and their perceptions of unmet service needs for care recipients.

Future research should examine how unmet needs and indicators of caregiver burden interact over time. Longitudinal studies are needed to clarify whether unmet needs contribute to burden, whether burden increases the likelihood of perceiving needs as unmet, or whether both processes occur simultaneously. Future studies could also examine whether social restrictions moderate the association between unmet needs and well-being. Service innovation studies are warranted, including pilot projects testing integrated packages of psychological support, education, episodic relief, and practical home-based assistance.

### Strengths and Limitations

A key strength of this study is the use of the interRAI-SCaN to directly assess caregivers’ functional abilities and caregiving experiences within a standardized assessment framework. The instrument also captures caregivers’ assessments of care recipient needs, allowing caregiver-reported support gaps to be examined alongside burden-related indicators.

This study has limitations. No a priori sample size calculation was performed, as a total population approach was used. Its cross-sectional design precludes causal inference, and reliance on self-reports may involve bias. Potential endogeneity cannot be ruled out, as some relevant variables were unavailable or exhibited limited variability. The dichotomization of the CWBI into excellent versus non-excellent well-being supported the study’s aim but reduced the outcome’s variation. The association with unmet need for psychological counseling should also be interpreted cautiously, as this need may be conceptually close to the psychological well-being outcome. The interRAI-SCaN focuses on caregivers and does not include detailed measures of care recipients’ health status; variables such as duration of caregiving and care intensity showed limited variability and were therefore excluded. Differences in recall periods across items may also have limited comparability across domains. Although valuable, the interRAI-SCaN warrants further validation. Findings are primarily generalizable to comparable urban home care nursing settings.

## Conclusion

This study indicates that selected unmet needs and burden-related indicators are associated with excellent caregiver well-being among informal caregivers. Caregiver-reported care-recipient unmet need for housing adaptation and caregiver unmet need for psychological counseling remained associated with lower odds of excellent caregiver well-being after burden-related indicators were added. Decreased social activities, caregiving-related stress, and multifaceted strain were also associated with lower odds of excellent well-being. These findings support the value of systematic caregiver assessment that addresses both support gaps and subjective caregiving strain. Addressing unmet needs may support caregiver well-being and support the sustainability of care systems in aging populations.

## Supplemental Material

Supplemental Material - Associations of Caregiver-Reported Unmet Needs and Burden-Related Indicators With Excellent Well-Being: A Cross-Sectional Study Supplemental Material for Associations of Caregiver-Reported Unmet Needs and Burden-Related Indicators With Excellent Well-Being: A Cross-Sectional Study by Sirrý Sif Sigurlaugardóttir, Sigurveig H. Sigurðardóttir, Thor Aspelund, Kristín Björnsdóttir, Magnus Jegermalm, Kjartan Ólafsson, and Ingibjörg Hjaltadóttir in INQUIRY: The Journal of Health Care Organization, Provision, and Financing.

Supplemental Material - Associations of Caregiver-Reported Unmet Needs and Burden-Related Indicators With Excellent Well-Being: A Cross-Sectional Study Supplemental Material for Associations of Caregiver-Reported Unmet Needs and Burden-Related Indicators With Excellent Well-Being: A Cross-Sectional Study by Sirrý Sif Sigurlaugardóttir, Sigurveig H. Sigurðardóttir, Thor Aspelund, Kristín Björnsdóttir, Magnus Jegermalm, Kjartan Ólafsson, and Ingibjörg Hjaltadóttir in INQUIRY: The Journal of Health Care Organization, Provision, and Financing.

Supplemental Material - Associations of Caregiver-Reported Unmet Needs and Burden-Related Indicators With Excellent Well-Being: A Cross-Sectional Study Supplemental Material for Associations of Caregiver-Reported Unmet Needs and Burden-Related Indicators With Excellent Well-Being: A Cross-Sectional Study by Sirrý Sif Sigurlaugardóttir, Sigurveig H. Sigurðardóttir, Thor Aspelund, Kristín Björnsdóttir, Magnus Jegermalm, Kjartan Ólafsson, and Ingibjörg Hjaltadóttir in INQUIRY: The Journal of Health Care Organization, Provision, and Financing.

Supplemental Material - Associations of Caregiver-Reported Unmet Needs and Burden-Related Indicators With Excellent Well-Being: A Cross-Sectional Study Supplemental Material for Associations of Caregiver-Reported Unmet Needs and Burden-Related Indicators With Excellent Well-Being: A Cross-Sectional Study by Sirrý Sif Sigurlaugardóttir, Sigurveig H. Sigurðardóttir, Thor Aspelund, Kristín Björnsdóttir, Magnus Jegermalm, Kjartan Ólafsson, and Ingibjörg Hjaltadóttir in INQUIRY: The Journal of Health Care Organization, Provision, and Financing.

Supplemental Material - Associations of Caregiver-Reported Unmet Needs and Burden-Related Indicators With Excellent Well-Being: A Cross-Sectional Study Supplemental Material for Associations of Caregiver-Reported Unmet Needs and Burden-Related Indicators With Excellent Well-Being: A Cross-Sectional Study by Sirrý Sif Sigurlaugardóttir, Sigurveig H. Sigurðardóttir, Thor Aspelund, Kristín Björnsdóttir, Magnus Jegermalm, Kjartan Ólafsson, and Ingibjörg Hjaltadóttir in INQUIRY: The Journal of Health Care Organization, Provision, and Financing.

Supplemental Material - Associations of Caregiver-Reported Unmet Needs and Burden-Related Indicators With Excellent Well-Being: A Cross-Sectional Study Supplemental Material for Associations of Caregiver-Reported Unmet Needs and Burden-Related Indicators With Excellent Well-Being: A Cross-Sectional Study by Sirrý Sif Sigurlaugardóttir, Sigurveig H. Sigurðardóttir, Thor Aspelund, Kristín Björnsdóttir, Magnus Jegermalm, Kjartan Ólafsson, and Ingibjörg Hjaltadóttir in INQUIRY: The Journal of Health Care Organization, Provision, and Financing.

## Data Availability

Data available from the corresponding author on reasonable request; ethical restrictions apply.*
