# Supplementary figures and images for: Associations of Caregiver-Reported Unmet Needs and Burden-Related Indicators With Excellent Well-Being: A Cross-Sectional Study
Source: Inquiry. 2026 Jul 6;63:00469580261466521. doi: 10.1177/00469580261466521 (PMC13342370; doi:10.1177/00469580261466521)

Supplementary Table 1: Flow diagram for study participants

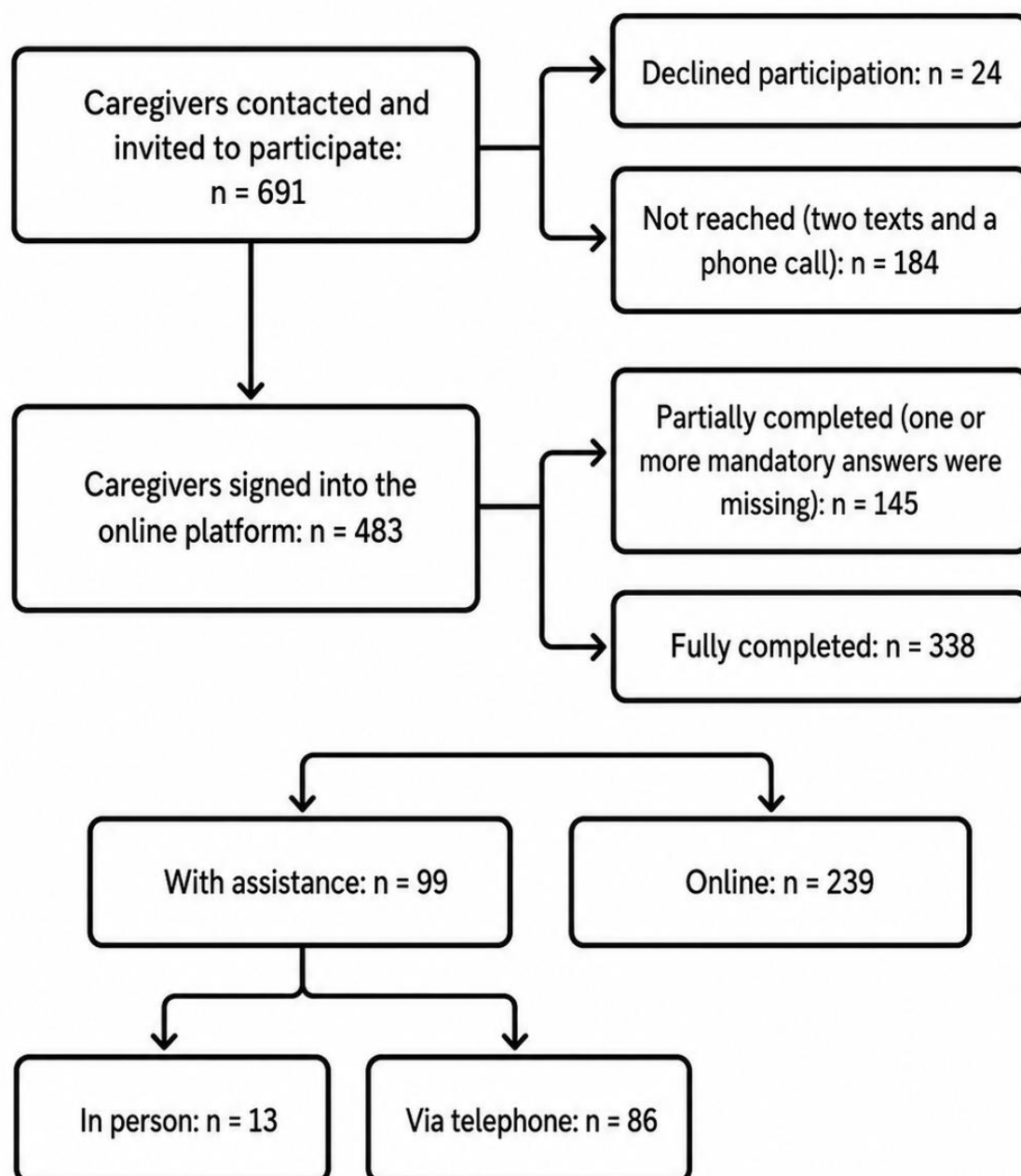

Supplement: Supplemental Material - Associations of Caregiver-Reported Unmet Needs and Burden-Related Indicators With Excellent Well-Being: A Cross-Sectional Study [file sj-pdf-1-inq-10.1177_00469580261466521.pdf]
