# Supplementary material for: Associations of Caregiver-Reported Unmet Needs and Burden-Related Indicators With Excellent Well-Being: A Cross-Sectional Study
Source: Inquiry. 2026 Jul 6;63:00469580261466521. doi: 10.1177/00469580261466521 (PMC13342370; doi:10.1177/00469580261466521)
Supplement: Supplemental Material - Associations of Caregiver-Reported Unmet Needs and Burden-Related Indicators With Excellent Well-Being: A Cross-Sectional Study [file sj-pdf-2-inq-10.1177_00469580261466521.pdf]

Supplementary table 2: Caregiver ADL ability in the last three days (n=350)

	Caregiver ADL ability in the last three days		
	I could do it all by myself	I would need some help from others	Others must do this for me
ADL activity	%	%	%
Meal preparation	85.2	10.8	3.7
Household chores	74.7	17.0	7.7
Managing finances	86.4	6.8	6.3
Managing medications	91.2	4.5	3.7
Shopping	83.5	7.7	8.2
Transportation	88.4	1.1	9.9
Bathing	92.3	5.4	1.7
