# Supplementary material for: Associations of Caregiver-Reported Unmet Needs and Burden-Related Indicators With Excellent Well-Being: A Cross-Sectional Study
Source: Inquiry. 2026 Jul 6;63:00469580261466521. doi: 10.1177/00469580261466521 (PMC13342370; doi:10.1177/00469580261466521)
Supplement: Supplemental Material - Associations of Caregiver-Reported Unmet Needs and Burden-Related Indicators With Excellent Well-Being: A Cross-Sectional Study [file sj-pdf-4-inq-10.1177_00469580261466521.pdf]

Supplementary Table 4: Significant associations between caregiver/care recipient characteristics and unmet needs (Chi-square tests)

**Outcome variable**	**χ^2 (df), p-value,**	**Interpretation**
Caregiver gender		
Mental health service	$\chi^2(1) = 3.88$, $p = 0.049$*	(female caregivers higher unmet need)
Psychological counselling	$\chi^2(1) = 8.52$, $p = 0.004$**	(female caregivers higher unmet need)
Episodic relief	$\chi^2(1) = 6.50$, $p = 0.011$*	(female caregivers higher unmet need)
Caregiver age		
Ordinary housework	$\chi^2(6) = 53.21$, $p < 0.001$***	(75+ highest need)
Managing finances	$\chi^2(6) = 19.61$, $p = 0.003$**	(75+ highest need)
Transportation	$\chi^2(6) = 21.61$, $p = 0.001$**	(75+ highest need)
Delivered meals	$\chi^2(9) = 43.57$, $p < 0.001$***	(50–64 highest unmet need)
Psychological counselling (support)	$\chi^2(2) = 24.40$, $p < 0.001$***	(under 49 highest unmet need)
Care recipient age		
Psychological counselling	$\chi^2(2) = 6.30$, $p = 0.043$*	(75–84 highest unmet need)
Physical rehabilitation	$\chi^2(2) = 6.58$, $p = 0.037$*	(75–84 highest unmet need)
Mental health service	$\chi^2(2) = 9.93$, $p = 0.007$**	(more unmet needs in 65–84, lower in 85+)
Marital status		
Housing adaptation	$\chi^2(1) = 5.47$, $p = 0.019$*	(married caregivers higher unmet needs)
Health education	$\chi^2(1) = 3.87$, $p = 0.049$*	(married caregivers higher unmet needs)
Relationship to care recipient		
Transportation assistance	$\chi^2(2) = 12.88$, $p = 0.002$**	(children/in-law higher unmet needs)
Delivered meals	$\chi^2(2) = 16.40$, $p < 0.001$***	(children/in-law higher unmet needs)
Ordinary housework	$\chi^2(2) = 46.15$, $p < 0.001$***	(spouses need more help)
Caregiver well-being (CWBI)		
Episodic relief	$\chi^2(1) = 36.30$, $p < 0.001$***	(more unmet needs when not excellent WB)
Psychological counselling	$\chi^2(1) = 48.86$, $p < 0.001$***	(more unmet needs when not excellent WB)
Housing adaptation	$\chi^2(1) = 29.76$, $p < 0.001$***	(more unmet needs when not excellent WB)
Health education	$\chi^2(1) = 21.99$, $p < 0.001$***	(more unmet needs when not excellent WB)
