# Supplementary material for: Associations of Caregiver-Reported Unmet Needs and Burden-Related Indicators With Excellent Well-Being: A Cross-Sectional Study
Source: Inquiry. 2026 Jul 6;63:00469580261466521. doi: 10.1177/00469580261466521 (PMC13342370; doi:10.1177/00469580261466521)
Supplement: Supplemental Material - Associations of Caregiver-Reported Unmet Needs and Burden-Related Indicators With Excellent Well-Being: A Cross-Sectional Study [file sj-pdf-5-inq-10.1177_00469580261466521.pdf]

Supplementary Table 5: Univariate logistic regression on background variables and needs

Background variables	**OR [CI]**
Female Caregiver	0.42 [0.25–0.71]*
Caregiver age	0.99 [0.07–1.01]
Married/partnered Caregivers	2.48 [1.42–4.33]*
Female Care Recipient	1.63 [1.04–2.55]*
Age of Care Recipient	1.00 [0.97–1.02]
Spouse of Care Recipient	0.48 [0.28–0.82]*
Child or child-in-law of Care Recipient	2.21 [1.34–3.62]*
Caregiver shares home with Care Recipient	0.44 [0.26–0.73]*
Caregiver functional ability	**OR [CI]**
Caregiver needs help with meal prep	0.42 [0.23–0.78]*
Caregiver needs help with ordinary housework	0.36 [0.22–0.59]*
Caregiver needs help with managing finances	0.46 [0.24–0.85]*
Caregiver needs help with managing medications	0.38 [0.17–0.82]*
Caregiver needs help with shopping	0.24 [0.13–0.43]*
Caregiver needs help with transportation	0.31 [0.16–0.61]*
Caregiver-reported care recipient unmet needs	**OR [CI]**
Assistance with personal care	0.38 [0.23–0.60]*
Assistance with household tasks	0.44 [0.27–0.70]*
Medical or nursing care	0.48 [0.30–0.76]*
Mental health service	0.45 [0.28–0.71]*
Delivered meals	0.54 [0.34–0.85]*
Daycare services outside of home	0.52 [0.33–0.81]*
End-of-life care	0.40 [0.17–0.96]*
Housing adaptation	0.21 [0.12–0.38]*
Aids and assistive devices	0.40 [0.25–0.64]*
Transportation assistance	0.56 [0.36–0.89]*
Caregiver support needs	**OR [CI]**
Carer support group	0.25 [0.15–0.42]*
Psychological counselling	0.19 [0.11–0.30]*
Health education	0.33 [0.20–0.53]*
Episodic relief from caregiving	0.24 [0.15–0.39]*

Odds Ratios (Exp(B)) with 95% Confidence Intervals in brackets. * Indicates statistical significance at $p < .05$.
