# Supplementary material for: Associations of Caregiver-Reported Unmet Needs and Burden-Related Indicators With Excellent Well-Being: A Cross-Sectional Study
Source: Inquiry. 2026 Jul 6;63:00469580261466521. doi: 10.1177/00469580261466521 (PMC13342370; doi:10.1177/00469580261466521)
Supplement: Supplemental Material - Associations of Caregiver-Reported Unmet Needs and Burden-Related Indicators With Excellent Well-Being: A Cross-Sectional Study [file sj-pdf-6-inq-10.1177_00469580261466521.pdf]

Supplementary Table 6: Multivariable logistic regression models for each predictor group

Background variables	**OR [CI]**
Female Caregiver	0.41 [0.23–0.72]*
Caregiver age	1.04 [1.00–1.07]*
Married/partnered Caregivers	3.04 [1.65–5.60]*
Female Care Recipient	1.21 [0.72–2.01]
Age of Care Recipient	0.95 [0.91–0.99]*
Spouse of Care Recipient	0.76 [0.13–4.52]
Child or child-in-law of Care Recipient	3.35 [1.14–9.82]*
Caregiver shares home with Care Recipient	0.49 [0.13–1.81]
Caregiver functional ability	**OR [CI]**
Needs help with meal preparation	0.89 [0.38–2.09]
Needs help with ordinary housework	0.52 [0.26–1.02]
Needs help with managing finances	2.08 [0.71–6.09]
Needs help with managing medications	1.36 [0.44–4.26]
Needs help with shopping	0.20 [0.07–0.59]*
Needs help with transportation	1.02 [0.33–3.10]
Caregiver-reported care recipient unmet needs	**OR [CI]**
Unmet needs for assistance with personal care	0.60 [0.34–1.09]
Unmet needs for housework	0.89 [0.49–1.61]
Unmet needs for mental health services	0.77 [0.44–1.33]
Unmet needs for delivered meals	0.88 [0.51–1.51]
Unmet needs for daycare services outside of the home	0.92 [0.53–1.57]
Unmet needs for end-of-life care	0.68 [0.26–1.79]
Unmet needs for housing adaptation	0.32 [0.17–0.62]*
Unmet needs for aids and assistive devices	0.68 [0.39–1.16]
Unmet needs for transportation assistance	1.14 [0.64–2.01]
Caregiver support needs	**OR [CI]**
Unmet need: Carer support group	0.79 [0.40–1.55]
Unmet need: Psychological counselling	0.30 [0.16–0.56]*
Unmet need: Health education	0.84 [0.46–1.52]
Unmet need: Episodic relief from caregiving	0.39 [0.23–0.67]*

Odds Ratios (Exp(B)) with 95% Confidence Intervals in brackets. * Indicates statistical significance at $p < .05$.
